# The 2021–2022 position of Brazilian Diabetes Society on diabetic kidney disease (DKD) management: an evidence-based guideline to clinical practice. Screening and treatment of hyperglycemia, arterial hypertension, and dyslipidemia in the patient with DKD

**DOI:** 10.1186/s13098-022-00843-8

**Published:** 2022-06-11

**Authors:** João Roberto de Sá, Erika Bevilaqua Rangel, Luis Henrique Canani, Andrea Carla Bauer, Gustavo Monteiro Escott, Themis Zelmanovitz, Marcello Casaccia Bertoluci, Sandra Pinho Silveiro

**Affiliations:** 1 Endocrinology Division, Escola Paulista de Medicina, UNIFESP, São Paulo, Brazil; 2 Nephrology Division, UNIFESP, São Paulo, Brazil; 3 Instituto Israelita de Ensino e Pesquisa Albert Einstein, Hospital Israelita Albert Einstein, São Paulo, Brazil; 4 Internal Medicine Department, Universidade Federal do Rio Grande do Sul (UFRGS), Porto Alegre, Brazil; 5 Endocrinology Division, Hospital de Clínicas de Porto Alegre (HCPA), Ramiro Barcelos, 2350—Prédio 12, 4º andar, Porto Alegre, RS Brazil

**Keywords:** Diabetes mellitus, Diabetic kidney disease, Diabetic nephropathy, Management, Treatment

## Abstract

**Background:**

Diabetic kidney disease is the leading cause of end-stage renal disease and is associated with increased morbidity and mortality. This review is an authorized literal translation of part of the Brazilian Diabetes Society (SBD) Guidelines 2021–2022. This evidence-based guideline provides guidance on the correct management of Diabetic Kidney Disease (DKD) in clinical practice.

**Methods:**

The methodology was published elsewhere in previous SBD guidelines and was approved by the internal institutional Steering Committee for publication. Briefly, the Brazilian Diabetes Society indicated 14 experts to constitute the Central Committee, designed to regulate methodology, review the manuscripts, and make judgments on degrees of recommendations and levels of evidence. SBD Renal Disease Department drafted the manuscript selecting key clinical questions to make a narrative review using MEDLINE via PubMed, with the best evidence available including high-quality clinical trials, metanalysis, and large observational studies related to DKD diagnosis and treatment, by using the MeSH terms [diabetes], [type 2 diabetes], [type 1 diabetes] and [chronic kidney disease].

**Results:**

The extensive review of the literature made by the 14 members of the Central Committee defined 24 recommendations. Three levels of evidence were considered: A. Data from more than 1 randomized clinical trial or 1 metanalysis of randomized clinical trials with low heterogeneity (I^2^ < 40%). B. Data from metanalysis, including large observational studies, a single randomized clinical trial, or a pre-specified subgroup analysis. C: Data from small or non-randomized studies, exploratory analyses, or consensus of expert opinion. The degree of recommendation was obtained based on a poll sent to the panelists, using the following criteria: Grade I: when more than 90% of agreement; Grade IIa 75–89% of agreement; IIb 50–74% of agreement, and III, when most of the panelist recommends against a defined treatment.

**Conclusions:**

To prevent or at least postpone the advanced stages of DKD with the associated cardiovascular complications, intensive glycemic and blood pressure control are required, as well as the use of renin–angiotensin–aldosterone system blocker agents such as ARB, ACEI, and MRA. Recently, SGLT2 inhibitors and GLP1 receptor agonists have been added to the therapeutic arsenal, with well-proven benefits regarding kidney protection and patients’ survival.

## Introduction

Diabetic kidney disease is the leading cause of entry into renal replacement therapy and is associated with increased morbidity and mortality [1, 2].

In 2007, the Kidney Disease Outcomes Quality Initiative (KDOQI) proposed the expression of diabetic kidney disease (DKD) in place of diabetic nephropathy (DN) to broaden the spectrum of forms of kidney disease in diabetes mellitus (DM), adding the non-albuminuric phenotype to the already described albuminuric phenotype [3, 4]. The use of the term DN has been suggested for the specific picture of elevated albuminuria followed by the subsequent progressive loss of renal function.

Traditionally, DKD was defined as a sequential evolution of stages where the onset would be characterized by glomerular hyperfiltration and renal hypertrophy, followed by a progressive increase in urinary albumin excretion (UAE) between 30 mg/day and 300 mg/day (formerly known as microalbuminuria) and subsequently by UAE greater than 300 mg/day or macroalbuminuria. We decided to keep this nomenclature of micro- and macroalbuminuria throughout the text to be faithful to the existing literature, since the vast majority still use these terms. In the more advanced phases, a progressive loss of glomerular filtration rate (GFR) starts, culminating in end-stage renal disease (ESRD) [5, 6]. However, in recent years it has been recognized that this evolution does not always happen, as there are patients who lose glomerular filtration without developing albuminuria [4, 7], a fact associated with multiple factors such as hypertension, dyslipidemia, obesity, and age [8].

## Methodology

The present review is a literal authorized translation of part of the 2021–2022 Brazilian Diabetes Society (Sociedade Brasileira de Diabetes—SBD) Guidelines. The methodology was published elsewhere in previous guidelines of SBD [9] and was approved by the internal institutional Steering Committee for publication.

In brief, the Brazilian Diabetes Society indicated 14 experts to constitute the Central Committee, designed to regulate methodology, review the manuscripts, and make judgments on degrees of recommendations and levels of evidence. SBD Renal Disease Department drafted the manuscript selecting key clinical questions to make a narrative review using MEDLINE via PubMed, using the best evidence available including high-quality clinical trials, metanalysis, and large observational studies related to Diabetic Kidney Disease diagnosis and treatment, by using the MeSH terms [diabetes], [type 2 diabetes], [type 1 diabetes] and [chronic kidney disease].

### Level of evidence

Three levels of evidence were considered: A. Data from more than 1 randomized clinical trial or 1 metanalysis of randomized clinical trials with low heterogeneity (I^2^ < 40%). B. Data from metanalysis, including large observational studies, a single randomized clinical trial, or a pre-specified subgroup analysis. C: Data from small or non-randomized studies, (cross-sectional, case–control, or experimental) exploratory analyses, or consensus of expert opinion.

### Degree of recommendation

A poll was sent to all expert panelists from the Renal Department and Central Committee for each defined recommendation. The frequency of responses was analyzed, and a degree of recommendation was obtained based on the following criteria: Grade I: when more than 90% of the participants agree; Grade IIa 75–89% of the panelists agree; IIb 50–74% of the panelists agree and III, when the greatest part of the panelist recommends against a defined treatment. The terminology used related to the four degrees of recommendations were: 1. IS RECOMMENDED; IIa. SHOULD BE CONSIDERED; IIb MAY BE CONSIDERED, and III: IT IS NOT RECOMMENDED.

### Definitions

**Fig. 1:**
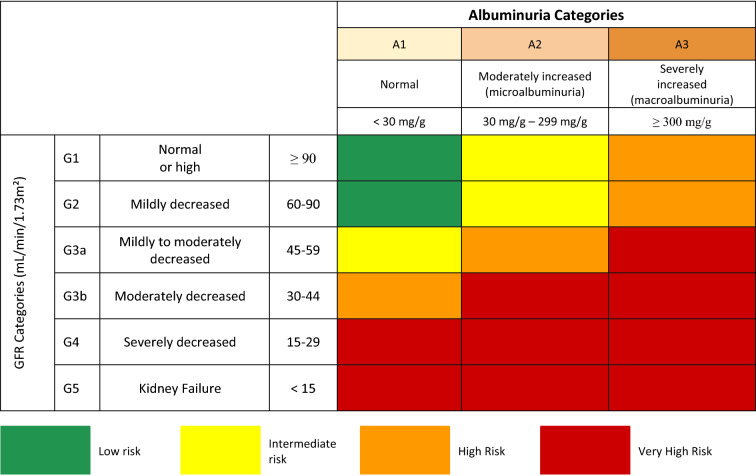
Stages of chronic kidney disease according to glomerular filtration rate (GFR) and albuminuria levels and risk rating for progression to end-stage kidney disease. Source: Adapted from KDIGO [11]

The SBD endorses the staging proposed by Kidney Disease Improving Global Outcomes (KDIGO) for DKD, which combines stages of renal function loss based on glomerular filtration rate (GFR) and urinary albumin excretion (UAE), using the two parameters in a complementary way (Fig. 1) [10].

### Albuminuria

Any abnormally elevated albuminuria test must be confirmed in two out of three samples collected in 3 to 6 months, due to the large daily variability [12, 13]. Factors such as fever, intense exercise, decompensated heart failure, severe hyperglycemia, urinary tract infection, and uncontrolled arterial hypertension can elevate UAE values [14]. The presence of asymptomatic bacteriuria does not significantly interfere with the result [15]. Albuminuria may regress in about 30% of patients, not necessarily related to therapeutic intervention [11, 16].

### Glomerular filtration rate (GFR) estimation

There are several formulas to estimate GFR. The one recommended by KDIGO is the CKD-EPI (Chronic Kidney Disease Epidemiology Collaboration), which, however, may underestimate the GFR in people with diabetes [10, 17–19]. The original 2009 CKD-EPI equation includes serum creatinine, age, gender, and race in the calculation of GFR. Recently, another race free equation was developed, the 2021 CKD-EPI equation, based on the doubt of the real need of the inclusion of the race factor present in the 2009 equation, which increased the final value in about 16%. These issues are still open to debate, but the 2021 CKD-EPI equation is at the present the recommended equation. These formulas are easily accessible on websites such as www.sbn.org.br and www.kidney.org or in apps, such as the official eGFR from the National Kidney Foundation.

## Recommendations

### DKD Screening

#### R1

The first screen for DKD IS RECOMMENDED to be at the diagnosis in T2DM, and after 5 years from diagnosis in people with T1DM, starting at 11 years of age.


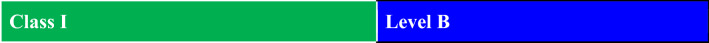


### Summary of evidence

UAE was determined [20] in 957 patients aged 5 years or older with type 1 diabetes mellitus (T1DM), with a prevalence of microalbuminuria of 22% and macroalbuminuria of 19%. A 37% prevalence of microalbuminuria was detected in adolescents between 15 and 18 years of age and no cases were detected in adolescents under the age of 15 years. In 119 individuals with 5 to 9 years of T1DM, a prevalence of microalbuminuria was found to be around 3%.

Regarding type 2 diabetes mellitus (T2DM), DKD screening should be performed at the time of DM diagnosis, as 7% of patients already have microalbuminuria at that time [7].

**Important note 1**: Screening in children and adolescents.

In children and adolescents with T1DM, screening can also be done at 2 to 5 years of disease duration and 11 to 17 years of age, considering the observed prevalence of 3% in a cohort study.

#### R2

IT IS RECOMMENDED to perform an annual screening of DKD with the measurement of albumin or albumin/creatinine ratio in a urine sample, together with the estimation of GFR with the serum creatinine-based CKD-EPI equation.


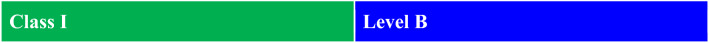


### Summary of evidence

Screening should be annual, measuring UAE and estimating GFR (eGFR) from serum creatinine [12, 21].

Urinary albumin should be measured in a random urine sample, for ease of collection [13]. There is no need to collect 24-h urine for screening, diagnosis, and follow-up of DKD [10, 22].

Albumin/creatinine ratio (mg/g) and the albumin concentration (mg/L) in the random urine sample show excellent correlation with the 24-h urinary measurement and can both be used [22, 23]. In a meta-analysis that analyzed 14 studies, sensitivities of around 85% and 87% were reported for albumin concentration and albumin/creatinine ratio, respectively; specificity was 88% for both for detection of microalbuminuria [24]. Considering the lower cost, the concentration of albumin seems to be advantageous [25]. It is argued that the albumin/creatinine ratio corrects for an eventual dilution/concentration of the urine. Therefore, considering that the diagnostic performance is similar, both can be used.

GFR and UAE are independent predictors of the course of kidney disease and risk of mortality, and therefore both should be evaluated in screening for DKD [26].

The recent characterization of non-albuminuric forms of DKD also reinforces the importance of adding the estimated GFR to the measurement of albuminuria [27, 28].

#### R3

IT IS RECOMMENDED that any abnormal test of the albumin/creatinine ratio (above 30 mg/g) or albumin concentration (above 30 mg/L) be confirmed in at least two out of three samples collected with an interval of three to six months because of the high daily variability.


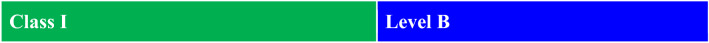


### Summary of evidence

Cutoff points for albumin in urine specimens (> 30 mg/g or > 30 mg/L) are derived from comparison with 24-h urine specimens, demonstrating adequate performance as a screening and diagnostic test [23, 24, 28, 29].

In a prospective evaluation, the albumin concentration ≥ 14 mg/L in a urine sample increased approximately threefold the risk of CV events (HR 3.25; 95% CI 1.43—7.38; p = 0.005) four times the risk of DKD (HR 4.3; 95% CI 2.22—8.32; p < 0.001) and five times the risk of death (HR 5.51; 95% CI 1.16—26, 22; p = 0.032), which indicates that even values ​​below the mentioned cutoff can predict cardiorenal outcomes [29].

An altered albuminuria test result should be confirmed in two out of three samples, collected in three to six months because of the large daily variability [12, 13]. If possible, assess in the absence of decompensated heart failure, uncontrolled hyperglycemia, and high blood pressure [14, 15].

**Important note 2:** Special situations.

In special situations, such as puberty, decompensated diabetes, and pregnancy, screening should be individualized and performed at shorter intervals.

**Important note 3**: Albuminuria.

Albuminuria above 14 mg/L suggests an increased cardiovascular and renal risk. However, there is no evidence from intervention studies for the prevention of cardiovascular and renal outcomes in this stage. It is suggested that follow-up should be more frequent when there are values ​​between 14 mg/L and 30 mg/L.

Some conditions suggest the need to rule out other nephropathies (Table 1).

**Table 1:**
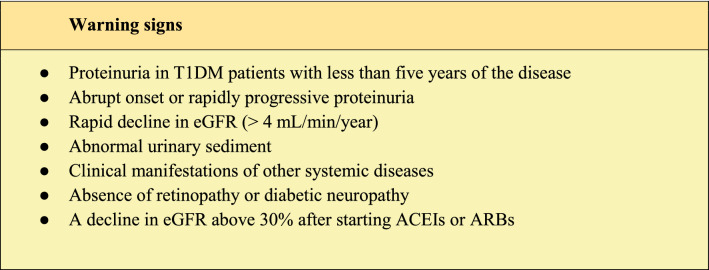
Warning signs to investigate another nephropathy

Source: Adapted from Gross et al. [21]. *ACEI* Angiotensin-converting enzyme inhibitors, *ARB* Angiotensin receptor blockers

## Prevention of DKD progression

Treatment of DKD aims to prevent progression to end-stage renal disease, intervene in cardiovascular events and prevent death. For this, risk factors for progression, such as hyperglycemia, hypertension, albuminuria, dyslipidemia, smoking, obesity, inadequate diet, and sedentary lifestyle, must be addressed.

Several studies have indicated that severe obesity (BMI > 40 kg/m^2^) enhances ESRD risk sevenfold [30]. Even a BMI > 25 kg/m^2^ was found to increase ESRD risk [30]. This effect is independent of the effects of hypertension and diabetes, the prevalence of which are increased in individuals with obesity. Obesity could cause an increased glomerular pressure and hyperfiltration [31], and adiponectin was suggested to link obesity to podocyte damage [32]. Weight reduction from bariatric surgery [33] or pharmacological treatment [34] has been associated with improved renal outcomes. In this context the strategies for the prevention and treatment of CKD include lifestyle changes and pharmacological approaches, including the promotion of exercise and dietary changes [35, 36].

### Treatment of hyperglycemia in DKD

#### R4

Intensive treatment of hyperglycemia in individuals with T1DM or T2DM IS RECOMMENDED for the prevention of DKD.


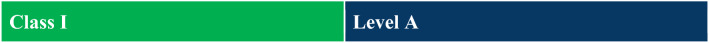


### Summary of evidence

The role of glycemic control in preventing the onset of albuminuria is well established in patients with T1DM and T2DM.

In the UKPDS (the United Kingdom Prospective Diabetes Study), intensive care in T2DM patients reduced HbA1c from 7.9% to 7.0%, with a 25% risk reduction in microvascular outcomes, although with no reduction in the risk of kidney outcomes specifically [37].

Other randomized clinical trials, whose main objective was to reduce macro and microvascular outcomes, were conducted, such as the ACCORD study (Action to Control Cardiovascular Risk in Diabetes) [38], ADVANCE (Action in Diabetes and Vascular Disease: Preterax and Diamicron Modified Release Controlled Evaluation) [39] and the VADT (Veterans Affair Diabetes Trial) [40].

In the ACCORD study [38], the incidence of macroalbuminuria was reduced by 29% in the intensive care group compared to conventional care (HbA1c 7.2% versus 7.6%).

In the ADVANCE study [39], the intensive care group reduced mean HbA1c from 7.3% to 6.5% and achieved a reduction in the incidence of new cases of microalbuminuria (HR 0.91 95% CI 0.85–0.98; p = 0.02) and the development of macroalbuminuria of 2.9% vs. 4.1% compared to the control group (HR 0.70 95% CI 0.57–0.85; p < 0.001).

In the VADT study [40], the intensive blood glucose treatment group achieved an HbA1c of 6.9%, while the control group maintained an HbA1c of 8.4%. In the intensive group, 10% of patients progressed from normoalbuminuria to micro- or macroalbuminuria, while in the control group the rate of progression was 14% (p = 0.03).

In the DCCT study (Diabetes Control and Complications Trial) [41], 1,441 individuals with T1DM were divided at baseline into primary prevention (albuminuria < 40 mg/24 h) and secondary prevention (albuminuria < 200 mg/24 h) cohorts, which were randomized for intensive or conventional treatment. The intensive treatment group achieved and maintained an average Hb1Ac of around 7%, while the control group maintained an HbA1c of around 9% over 6.5 years. With the two cohorts combined, intensive care reduced the incidence of microalbuminuria by 39% (95% CI 21–52%) and the occurrence of macroalbuminuria by 54% (95% CI 19–74%).

The Epidemiology of Diabetes Interventions and Complications (EDIC) study [42] was an observational extension of the DCCT that extended the results for up to 11 years. At the end of the study, the prevalence of microalbuminuria and macroalbuminuria in individuals with T1DM continued to be higher in the conventional treatment group compared to the intensive control group, respectively: HR 0.62 (0.39–0.97), p = 0. 04 and HR 0.58 95% CI 0.37–0.91), p = 0.02.

#### R5

Intensive control of hyperglycemia IS RECOMMENDED in individuals with DM to reduce albuminuria.


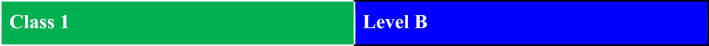


### Summary of evidence

The effect of intensive glycemic control on the decline in GFR and progression to macroalbuminuria in patients with microalbuminuria is not clearly defined. Evidence is derived from subgroup analyses. In this population, maintaining HbA1c below 7% appears to have only a mild long-term effect in delaying progression to end-stage renal failure [37, 41, 43, 44].

### Type 2 DM (T2DM)

The ADVANCE study [39] randomized 11,140 T2DM patients undergoing intensive glycemic control using gliclazide MR to search for 6.5% or lower HbA1c. In the study, 27% of participants had microalbuminuria, 3.6% had macroalbuminuria, and serum creatinine was initially normal (0.97–1.05 mg/dL). The primary composite endpoint included macrovascular endpoints and a composite microvascular endpoint, including onset or worsening of nephropathy, doubling of creatinine, need for replacement therapy, or death from kidney disease. At the end of five years of follow-up, there was only a tendency towards a reduction in the need for renal replacement therapy or death from renal causes (HR 0.64, 95% CI 0.38–1.08).

The ADVANCE ON [45] trial was a six-year observational extension of the ADVANCE [32] trial after completion. Participants received no intervention, and the HbA1c differences observed between groups at the end of ADVANCE disappeared. Of the original ADVANCE participants, 8,494 participated in the post-trial phase. With the cumulative time of the two studies amounting to 10 years to 11 years, it was observed that there was a significant cumulative benefit in the progression to end-stage renal disease (HR 0.54, 95% CI 0.34–0.85, p = 0.007).

In turn, the ACCORD study [38], in a subanalysis of microvascular outcomes, showed no difference in the effect of intensive blood-glucose treatment on the development of the combined outcome: renal failure, kidney transplantation, or creatinine increase: HR 0.95 (0.73–1.24).

In the VADT study [40], intensified treatment of hyperglycemia at the end of 5.6 years of follow-up (HbA1c 6.9% versus 8.4%) reduced the sequential progression from normoalbuminuria to microalbuminuria and macroalbuminuria. At the end of the study, 5.1% progressed to micro and macroalbuminuria in the control group, while only 2.9% progressed to the intensive group (p = 0.04).

Taken together, the results of these studies suggest that achieving HbA1c values below 7% has little effect in delaying the progression of kidney disease in patients with T2DM and established DKD. Furthermore, the protective action against progression to renal failure would only be observed after long periods of improvement in glycemic control.

The STENO 2 [43] Study was a randomized clinical trial conducted with 160 T2DM patients with microalbuminuria, with a follow-up of 7.8 years, aiming to evaluate whether intensive glycemic control associated with the control of other risk factors would affect micro and macrovascular outcomes. The intensified treatment group received multiple interventions, comprising ACEI, acetylsalicylic acid and lipid-lowering drugs, and intensive glycemic control (HbA1c 7.9% versus 9%). Intensive treatment of glycemia associated with the control of hypertension, control of dyslipidemia, and cessation of smoking revealed an important beneficial effect of the treatment on the loss of renal function assessed by eGFR and including a reduction in albuminuria. Although it was not possible to individualize the isolated effect of blood glucose reduction on renal outcomes, the study demonstrated the importance and need for controlling several risk factors, including blood glucose.

### Type 1 DM (T1DM)

In patients with T1DM, the DCCT study [44] did not find a reduction in the progression from micro to macroalbuminuria in patients who had microalbuminuria at baseline. However, the DCCT was not powerful enough to demonstrate this benefit, since only 73 patients initially had microalbuminuria.

Regarding mortality, a meta-analysis of randomized controlled trials (RCTs) has shown that intensive glycemic control in patients with T1DM does not reduce overall mortality or microvascular complications, including DKD [46].

A prospective observational study followed 349 T1DM patients from the Joslin Clinic, USA, with proteinuria (DKD stages 1 to 3) for up to 15 years. The group with better glycemic control during the observation period had a smaller drop in eGFR and a lower prevalence of end-stage renal disease (29%), compared to patients who maintained worse glycemic control (42%). A decrease in HbA1c by one point was associated with a 24% protection for the evolution of end-stage renal disease [47].

**Important note 4:** Target of HbA1c in advanced DKD.

It should be considered that very low or very high values of HbA1c are associated with negative outcomes in patients with DKD. In an observational study that evaluated 23,296 patients with DM and eGFR <60 mL/min, HbA1c values >8% and <6.5% were related to higher mortality [48]. This U-shaped mortality curve associated with HbA1c was also demonstrated in 9,000 patients with DM on hemodialysis for HbA1c values <7.0% and >7.9% [49]. A meta-analysis with ten studies, including 83,684 participants with DM on dialysis, concluded that individuals with HbA1c ≤ 5.4% or ≥ 8.5% had an increased risk of mortality [50]. In patients with DKD in stages 4-5, the glycated hemoglobin goal should be individualized as a function of the increased risk of hypoglycemia. Thus, in patients with advanced or terminal DKD, the best available evidence suggests that an HbA1c above 7.0% is adequate, but up to a maximum of 8% to 8.5% [36, 50].

### Treatment of hyperglycemia in mild to moderate DKD with GFR > 30 mL/min/1.73 m^2^

**Fig. 2:**
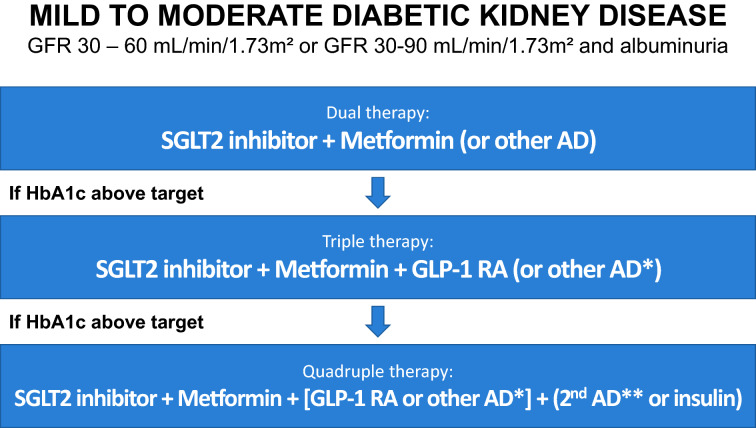
Algorithm for the treatment of hyperglycemia in patients with T2DM and DKD with GFR between 30 and 60 mL/min/1.73 m^2^ or between 30 and 90 mL/min/1.73 m^2^ with albuminuria. AD: Oral antidiabetic. Source: Adapted from Bertoluci et al. [51]. SGLT2: sodium-glucose cotransporter 2; GLP1: glucagon-like peptide 1

Figure 2 describes the Brazilian Society of Diabetes (SBD) proposal for managing hyperglycemia in mild to moderate DKD.

#### R6

In the treatment of T2DM and DKD with a GFR of 30-60 mL/min/1.73 m^2^ or albuminuria >200 mg/g, the use of SGLT2 inhibitors is RECOMMENDED to reduce progression to end-stage renal disease and death.


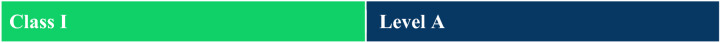


### Summary of evidence

Randomized clinical trials such as CREDENCE [52]—(Canagliflozin and Renal Events in Diabetes with Established Nephropathy Clinical Evaluation) and DAPA-CKD [53, 54] (Dapagliflozin and Prevention of Adverse Outcomes in Chronic Kidney Disease) evaluated sodium-glucose cotransporter 2 (SGLT2) inhibitors in patients with T2DM and DKD, proving the reduction of renal outcomes, such as progression to advanced kidney disease, the need for dialysis and renal death. In these studies, the inclusion criteria were GFR 25-75 mL/min/1.73m^2^ and albuminuria 200-5000 mg/g (DAPA CKD) and GFR 30-90 mL/min/1.73m^2^ with albuminuria 300-5000 mg/g (CREDENCE).

The EMPA-REG OUTCOME study (Empagliflozin Cardiovascular Outcome Event Trial in Type 2 Diabetes Mellitus Patients) [55, 56], using empagliflozin, studied renal outcomes in individuals with T2DM as a secondary outcome and observed a 38% reduction in microalbuminuria, 44 % reduction in the number of patients who doubled creatinine at follow-up time and a 55% reduction in patients requiring renal replacement therapy.

The CANVAS study (Canagliflozin Cardiovascular Assessment Study) [57] used canagliflozin in individuals with T2DM and demonstrated benefits in the progression of albuminuria, the need for renal replacement therapy, and the reduction of death from renal causes.

In the DECLARE-TIMI 58 [58] (Dapagliflozin Effect on Cardiovascular Events) study, dapagliflozin reduced the composite event of significant eGFR loss, progression to dialysis, and renal death by 47%.

**Important Note 5:** SGLT2 Inhibitors.

SGLT2 inhibitors are associated with a markedly increased risk of a genital infection (RR 3.56, 95% CI 2.84 - 4.46) and a mildly increased risk of urinary tract infection (RR 1.06, 95%CI 1.01-1.12). [59] In the CANVAS study [57], canagliflozin was associated with a higher incidence of bone fractures and lower limb amputations, although other studies, such as CREDENCE [54], have not confirmed these findings. There are reports of an increased incidence of Fournier's gangrene (perineal necrotizing fasciitis), but this association was not observed in the DECLARE-TIMI study 58 [58]. The occurrence of “euglycemic” ketoacidosis is also rare and is associated with insulinopenia [59].

#### R7

In patients with T2DM and DKD with GFR >30 mL/min/1.73 m^2^ , the combination of SGLT2 inhibitors with another antidiabetic drug, preferably metformin, SHOULD BE CONSIDERED to optimize glycemic control and potential reduction of cardiovascular risk, considering the limitations determined by glomerular filtration.


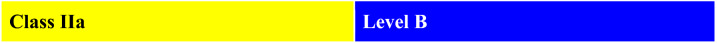


### Summary of evidence

In a sub-analysis evaluating patients using metformin from the TREAT study (Trial to Reduce Cardiovascular Events with Aranesp [darbepoetin-alpha] Therapy) [60], cardiovascular and renal outcomes after 4 years of follow-up were compared among 3,447 patients with T2DM who were not using metformin and 591 using metformin, of which 386 had stage 3b or more advanced DKD. Metformin use was associated with a lower risk of overall mortality (HR 0.49; 95% CI, 0.36–0.69), cardiovascular death (HR 0.49; 95% CI, 0.32–0.74) and composite cardiovascular outcome (HR 0.67; 95% CI, 0.51–0.88), although there was no evidence of specific renal benefits. Two cases of lactic acidosis were recorded, confirming the rare occurrence of this event. These data suggest that metformin does indeed appear to be safer than previously described, in addition to reducing mortality and cardiovascular events in patients with advanced DKD.

A recent Asian retrospective cohort study, involving 10,426 T2DM patients with DKD, confirms that the use of metformin was associated with lower overall mortality, with HR 0.65 (95% CI 0.57–0.73; p < 0.001) and further demonstrating reduced progression to end-stage renal disease, with HR 0.67 (95% CI 0.58 − 0.77; p < 0.001). Metformin did not increase the risk of lactic acidosis (HR 0.92; 95% CI 0.668–1.276; p = 0.629) [61].

**Important note 6**: Metformin adjustment for GFR.

In individuals with DKD and GFR between 30 and 45 mL/min/1.73 m^2^, it is recommended to reduce the dose of metformin, which should be limited to a dose of 1 g per day to minimize the risk of lactic acidosis. Metformin should not be use for GFR < 30 mL/min/1.73 m^2^. 

#### R8

In T2DM patients with DKD and GFR > 30 mL/min/1.73 m 2 , the use of GLP-1 receptor agonists (GLP-1 RA) SHOULD BE CONSIDERED to reduce albuminuria.


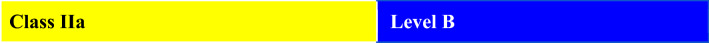


### Summary of evidence

Cardiovascular safety studies show that the use of GLP-1 RA, both daily [62] and weekly [60–62], is associated with decreased composite renal outcome (progression of albuminuria, doubling of creatinine, and death from renal cause). However, what led to protection was the reducing effect of albuminuria, reaching 50%, with a small effect on eGFR and other outcomes.

A meta-analysis [63] suggests that the effect in reducing albuminuria is class-related. However, it is important to emphasize that the studies were not designed to assess the renal effects, with albuminuria being a secondary outcome.

A real-life study with more than 38,000 patients using GLP-1 RA, compared to DPP4 inhibitors, suggests a benefit in hard outcomes, with a 24% decrease in renal death, hospitalization for renal events, or the initiation of renal replacement therapy.

### Treatment of hyperglycemia in severe DKD with GFR < 30 mL/min/1.73 m^2^

**Fig. 3:**
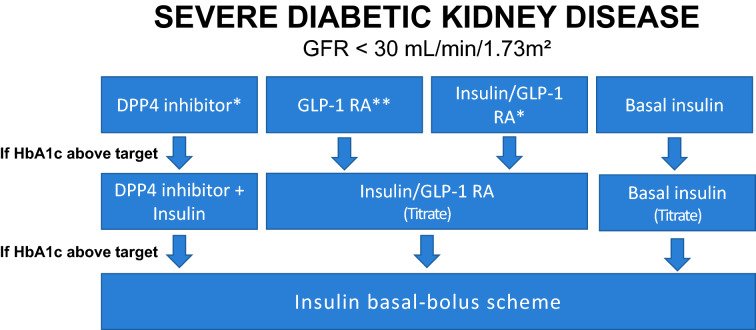
Management of hyperglycemia in severe DKD. Source: Adapted from Bertoluci et al. [64]. *Dose adjustment required, except for linagliptin. **Only if GFR > 15 mL/min/1.73 m^2^

Figure 3 shows the algorithm suggested by the SBD for the management of hyperglycemia in severe DKD with a GFR < 30 mL/min/1.73 m^2^.

#### R9

In individuals with T2DM and DKD with eGFR <30 mL/min/1.73 m^2^ , with HbA1c above the target, insulin treatment SHOULD BE CONSIDERED as a priority to improve glycemic control.


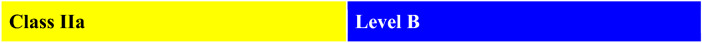


Glucose and insulin metabolism are greatly altered in patients with advanced kidney disease. There is a great risk of hypoglycemia because of reduced renal gluconeogenesis, reduced renal clearance and degradation of insulin, increased glucose uptake in dialysis, impaired hormonal counter-regulation, and nutritional deprivation. Therefore, the use of insulin with careful titration should always be thought of as the main choice for blood glucose control [65].

Regarding long-acting analogs, insulin Glargine is safe and effective in T2DM patients with advanced CKD, with a stable half-life and a longer duration of action. In a small, non-randomized study, 89 patients with T2DM and CKD (mean GFR 34.1 ± 11.5 mL/min/1.73 m^2^) with poorly controlled or that had frequent hypoglycemia with oral agents or NPH insulin, received insulin Glargine at bedtime, starting with 0.1 U/kg and later being titrated. In four months, HbA1c decreased from 8.4% ± 1.6 to 7.7% ± 1.2 (p < 0.001), without affecting the BMI and with no adverse event records [66].

A small crossover study randomized 34 T2DM patients with kidney disease stages 3 and 4 to insulin Glargine U100 or NPH. After 24 weeks, HbA1c was reduced with Glargine (− 0.91%; p < 0.001), but there was no benefit with NPH (0.23%; p = 0.93). In addition, the incidence of nocturnal hypoglycemia was three times lower with Glargine (p = 0.047) [67].

Regarding the long-acting analog Degludec, a small observational, retrospective, open-label study lasting 36 weeks evaluated its use in 36 patients with T2DM and eGFR < 45 mL/min/1.73 m^2^. With the switch from Detemir or Glargine (U100 or U300) to Degludec, the prevalence of mild hypoglycemia decreased from 78 to 34.2%, and that of severe hypoglycemia, from 8 to 1.3% [68].

The DEVOTE study (Dedicated CV outcomes trial) [69], involving 7637 participants with T2DM and high CV risk, demonstrated the CV safety of Degludec compared to Glargine-U100. Most patients (85.2%) had established CVD or CKD or both at baseline. There was a statistically significant 40% reduction in severe hypoglycemia with Degludec versus Glargine-100 (4.9 vs. 6.6%; RR 0.60, p < 0.001), with similar glycemic control.

A sub-analysis of the BRIGHT study [70] showed a greater reduction in HbA1c with Glargine U-300 compared to Degludec in the eGFR group < 60 mL/min/1.73 m^2^ with no difference in the incidence of hypoglycemia. The BRIGHT study was one 24-week, open-label, multicenter study with T2DM patients, who randomized to nocturnal Glargine-U300 (n = 466) or Degludec-U100 (n = 463) with 24 week follow-up, with the main outcome being reduced HbA1c.

#### R10

In patients with T2DM and DKD with a GFR of 15-30 mL/min/1.73 m^2^ , and HbA1c above target, DPP-4 inhibitors, some sulfonylureas (glipizide and gliclazide) and GLP-1 RA MAY BE CONSIDERED to improve glycemic control.


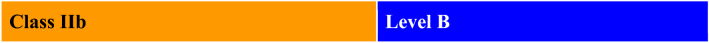


### Summary of evidence

#### Sulfonylureas

Concerning sulfonylureas, glipizide and gliclazide are completely metabolized in the liver, generating inactive metabolites. The renal function does not affect their clearance or half-life, yet they should be used with caution, and dose titration is recommended when estimated GFR (eGFR) is less than 30 mL/min/1.73 m^2^ [71].

A 54-week randomized clinical trial, with 129 T2DM patients over 30 years of age, with end-stage kidney disease on dialysis and 7–9% HbA1c, compared glipizide and sitagliptin. The group of 64 patients using sitagliptin 25 mg/day reduced baseline HbA1c by 0.72% vs 0.87% in the 65 patients who received glipizide 2.5 mg/day. The number of symptomatic hypoglycemia was not significantly different between the two groups (6.3% with sitagliptin, and 10.8% with glipizide). There was no severe hypoglycemia in the sitagliptin vs 5 episodes in the glipizide group, as these values were similar (difference of 7.8%; 95% CI − 17.1–− 1.9%) [72]. The treatment of T2DM patients on hemodialysis is safe with both glipizide and sitagliptin if their doses are adjusted.

#### DPP-4 inhibitors

In a non-randomized six-month trial, monotherapy with linagliptin (5 mg/day) in 21 patients with T2DM on hemodialysis reduced the glycated albumin (GA) from 21.3% ± 0.6% to 18.0% ± 0.6% throughout the treatment period, with no change in body weight. None of the patients had hypoglycemia [73].

In a sub-analysis of the SAVOR-TIMI trial 53 [74], 336 patients with severe renal impairment (eGFR < 30 mL/min/1.73 m^2^) were randomized to receive saxagliptin or placebo. After a mean duration of two years, saxagliptin did not change the relative risk of hospitalization for heart failure when compared to placebo, regardless of renal function (p = 0.19 for interactions). The median HbA1c at one year was lower when compared to placebo in saxagliptin-treated patients with severe renal impairment (7.1 vs. 7.7%, p = 0.002). At least one adverse event occurred in 152 patients (88%) treated with saxagliptin.

Estimated GFR must be considered regarding the use of medications for glycemic control in DKD. Table 2 shows the dose adjustments of the antidiabetic medications according to the stage of DKD [75] (Table 3).

**Table 2:**
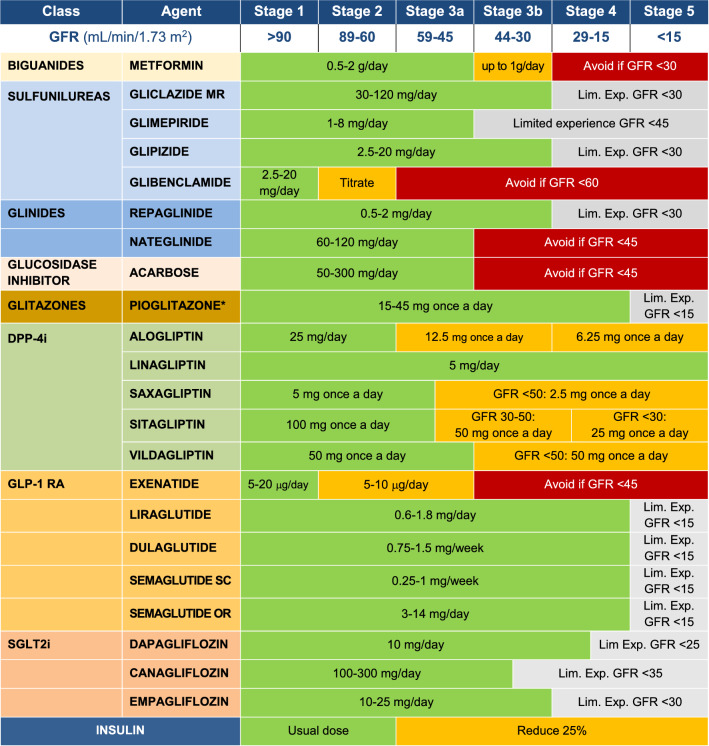
Antidiabetic agents with dose adjustments for renal function

^*^Pioglitazone, although it can be used across the entire spectrum of GFR, is associated with an increased risk of decompensated heart failure and fractures, and the potential risk–benefit should be evaluated. SU: sulfonylureas; Lim. Exp.: limited experience; GFR: Glomerular filtration rate; Source: Adapted from Escott et al. [75]

**Important note 8**: Options beyond insulin in severe DKD.

- DPP-4 inhibitors should be dose-adjusted according to GFR (see Table 2), except linagliptin, that does not need adjustment, and they should not be combined with GLP-1 RA.
- GLP-1 RA, alone or in fixed combination with insulin can only be used when GFR is above 15 mL/min/1.73 m^2^.
- Sulfonylureas (gliclazide and glipizide) can be used in severe DKD, but with caution and with dose reduction.

### Treatment of hyperglycemia in DKD on dialysis patients

#### R11

In individuals with T2DM on dialysis and HbA1c above the target, IT IS RECOMMENDED the use of insulin as a priority.


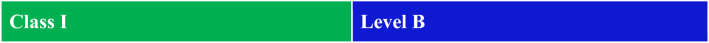


### Summary of evidence

Therapeutic individualization is very important in patients with DKD, especially in those with stages 4 and 5, because of the increased risk of hypoglycemia. Overall, insulin analogs appear to have a lower risk of hypoglycemia and better postprandial control compared to human insulin [76, 77].

Regarding basal insulins, glargine (U100 and U300), degludec, and detemir are stable and effective, but there are few studies in this population (patients with DKD), indicating that they are associated with a reduction in hypoglycemia, especially degludec when compared to detemir and glargine U100 [77, 78].

Concerning ultra-fast insulins, glulisine and aspart do not show pharmacokinetic differences in patients with severe CKD [79, 80]. As for insulin lispro, in turn, there may be a need for dose reduction [80].

**Important note 9**: Insulin in peritoneal dialysis.

The use of insulin in the dialysis solution in patients undergoing peritoneal dialysis is uncommon, and the reasons are the greater chance of peritoneal bacterial infection, use of higher doses, and higher occurrence of hepatic steatosis. Therefore, further studies are needed to evaluate the beneficial effect of this route, so that the subcutaneous route is preferably indicated [81, 82].

#### R12

In DM patients on dialysis, the use of insulin regimens based on hemodialysis time and pre-and post-dialysis blood glucose levels is RECOMMENDED, requiring a reduction of at least 25% of the dose of fast or ultra-fast insulin given just before the meal before dialysis.


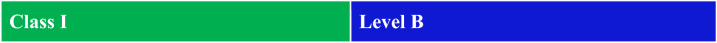


**Fig. 4:**
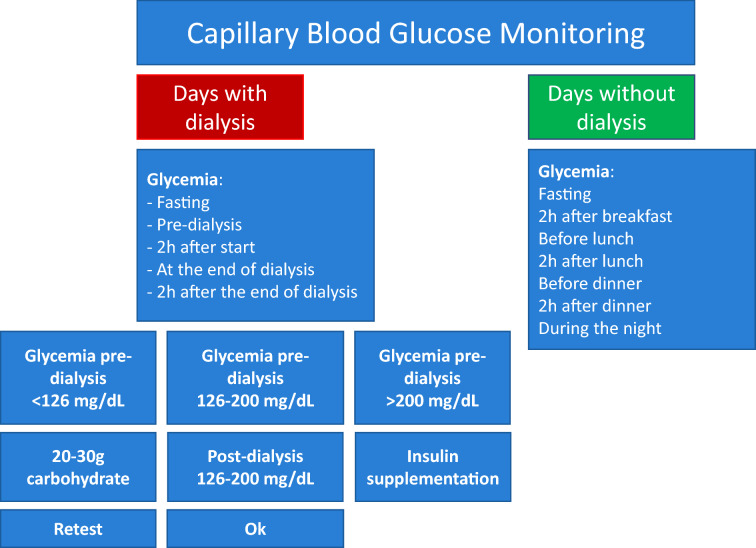
Recommendation for checking capillary blood glucose on days with and without dialysis. Adapted from Escott et al. [75]

In general, dialysis patients use two insulin regimens, one for the procedure day and one for the non-procedural days. The insulin regimen should be individualized, when in use, based on the time of hemodialysis and blood glucose values (Fig. 4).

A reduction of at least 25% of the dose of rapid or ultra-rapid insulin given in the meal before dialysis is required (Fig. 4).

**Important Note 10**: Insulin analogs in dialysis.

Insulin analogs should be applied after dialysis due to the possibility of insulin adsorption by the dialysis membrane.

**Important Note 11**: DPP-4 inhibitors in dialysis.

DPP-4 inhibitors can be considered for use in T2DM patients on dialysis with caution and dose reduction, as they are effective and likely to be safe. Since they have been tested in small studies, however, their long-term safety, especially regarding hypoglycemia, must be confirmed in larger studies.

### Treatment of hypertension in DKD

#### R13

Intensive treatment of hypertension is RECOMMENDED due to the cardiovascular benefits and the evolution of DKD.


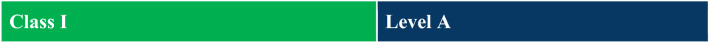


### Summary of evidence

Breakthrough studies, such as that by Parving [83], showed a reduction in UAE by lowering BP with captopril.

A meta-analysis of 40 studies with 100,354 participants (70% with T2DM subgroups and 30% with T2DM exclusively) demonstrated that a 10 mmHg reduction in systolic blood pressure was associated with a lower risk of mortality (RR 0.87; 95% CI 0. 78–0.96), of cardiovascular events (RR 0.89, 95% CI 0.83–0.95), of coronary heart disease (RR 0.88; 95% CI, 0.80–0.98); of stroke (RR 0.73 95% CI, 0.64–0.83]; of albuminuria (RR 0.83 95% CI 0.79–0.87); and of retinopathy (RR 0.87 95% CI 0.76–0.999). However, no reduction in the progression of DKD to ESRD was observed. The greatest benefit was among patients with baseline blood pressure > or = 140 mmHg. An additional reduction below 130 mmHg reduced the risks of stroke, retinopathy, and albuminuria [84].

#### R14

A blood pressure goal < 130/80 mmHg IS RECOMMENDED for patients with DKD who can reach this goal without side effects.


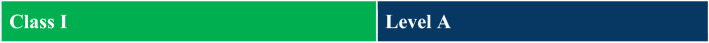


In proteinuric T2DM patients, the RENAAL study (Reduction of Endpoints in NIDDM with Angiotensin II Antagonist Losartan) demonstrated that achieving systolic pressure < 130 mmHg slowed the progression of DKD and postponed the need for dialysis [85].

Regarding the lower blood pressure limit to be reached in DKD patients, some studies, including the IDNT (Irbesartan Diabetic Nephropathy Trial), show that blood pressure ≤ 120/80 mmHg were associated with an increase in cardiovascular events [86].

Therefore, for patients with DKD, a blood pressure target of around 130/80 mmHg is suggested, which is in line with other guidelines.

In a meta-analysis of 19 studies including 44,989 participants (6,960 with DM), 2,496 cardiovascular events were observed after 3.8 years (1.0 to 8.4 years). In the intensive treatment group, the mean pressure was 133/76 mmHg vs. 140/81 mmHg in the less intensive group. In the intensive group, it was observed a 14% reduction in the RRs of cardiovascular events (95% CI 4—22%), 13% in acute myocardial infarction (95% CI 0—24%), 22% in stroke (95% CI 10—32%), 10% in albuminuria (95% CI 3–16%) and 19% reduction in retinopathy progression (95% CI 0—34%). The benefits were evident even in patients with a baseline blood pressure < 140 mmHg, more evident in the groups with vascular disease, kidney disease, and diabetes. However, there was no benefit for heart failure, cardiovascular/total death, or end-stage renal disease [87].

#### R15

A blood pressure goal < 130/80 mmHg IS RECOMMENDED for adult patients with DM and increased risks of stroke and atherosclerotic cardiovascular disease.


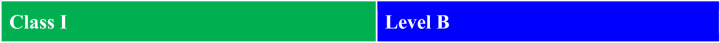


### Summary of evidence

The ADA recommends a blood pressure target < 130/80 mmHg for individuals at high cardiovascular risk (with atherosclerotic cardiovascular disease (ASCVD), or with ASCVD risk ≥ 15% in ten years), based on the risk analysis of previous studies [88, 89].

In a recent meta-analysis of individual participants data including 344,716 subjects from 48 RCTs (approximately 30% with DM), it was shown that a 5 mmHg reduction in systolic blood pressure reduced the risk of major CV events by 10% (fatal and non-fatal stroke, fatal and non-fatal AMI, ischemic heart disease and heart failure with death and hospitalization). This reduction was independent of previous CVD diagnosis and blood pressure levels, with a benefit even for normal blood pressure [90].

#### R16

It is RECOMMENDED to use angiotensin-converting enzyme inhibitors (ACEI) or angiotensin II receptor blockers (ARBs) for patients with albuminuria, to reduce kidney disease progression, regardless of blood pressure levels.


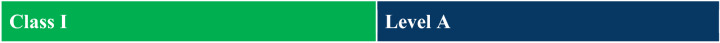


### Summary of evidence

Renin–angiotensin–aldosterone system (RAAS) blockers (ACEI and ARBs) reduce ACR and the progression of DKD to more advanced stages, regardless of the blood pressure effect [85, 91, 92].

In a meta-analysis of 1,028 studies of the effect of RAAS blockers on renal outcomes, 24 studies met the inclusion criteria (20 with ACEI and 4 with ARB). ACE inhibitors were associated with a trend towards a reduction in end-stage renal failure (RR 0.70; 95% CI 0.46–1.05), and the use of ARB reduced the risk (RR 0.78; CI 95% 0.67–0.91). Both reduced the risk of doubling creatinine (RR 0.71; 95% CI 0.56–0.91 for ACEI and RR 0.79; 95% CI 0.68–0.91 for ARB), but neither reduced the mortality [93].

Another meta-analysis on RAAS blocker, both ACEI, and ARBs, showed a reduction in albuminuria in T1DM and T2DM patients with microalbuminuria, and a reduction in the progression to macroalbuminuria, but not for end-stage renal failure or mortality [94].

The DETAIL study (Diabetics Exposed to Telmisartan and Enalapril) [95] showed an equivalent effect of the two classes of drugs in people with T2DM and micro-and macro-albuminuric DKD.

Another meta-analysis of 100 studies with data from 22,365 patients with DKD, mainly T2DM, showed no difference between ACEI and ARBs in preventing end-stage renal disease and doubling creatinine, as well as in reducing albuminuria [96].

The use of an ACEI or ARB is recommended for all patients with increased UAE, regardless of BP values [93, 97].

**Important Note 11**: RAAS Blockers.

After starting RAAS blockers, serum creatinine may increase by up to 30%. In this situation, the drug should not be discontinued, as this response is associated with the preservation of renal function, even in patients with baseline serum creatinine above 1.4 mg/dL. Creatinine elevations greater than 30%, in turn, should raise suspicion of renal artery stenosis [ 21].

#### R17

Combination therapy with ACEI and ARB IS NOT RECOMMENDED, due to the increased risk of hyperkalemia, worsening of renal function, orthostatic hypotension, and syncope.


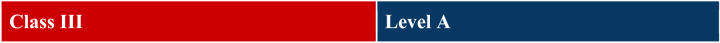


### Summary of evidence

The VA-NEPHRON D study evaluated the use of losartan (100 mg/day) in T2DM patients with at least 300 mg/g albuminuria and eGFR 30–89.9 mL/min/1.73 m^2^, with randomization to add lisinopril (10–40 mg/day) or placebo. The study was stopped early owing to safety concerns. From 1,448 patients with a 2.2-year follow-up, there were 152 events in the monotherapy group and 132 in the combination (HR 0.88; 95% CI, 0.70–1.12; p = 0.30). There was no reduction in mortality or cardiovascular events. However, there was an increased risk of hyperkalemia (6.3 events per 100 person/year vs. 2.6 events with monotherapy, p < 0.001) and of acute kidney injury (12.2 events vs. 6.7 events per 100 person/years, p < 0.001) [98].

#### R18

The use of mineralocorticoid receptor antagonists SHOULD BE CONSIDERED for blood pressure control and renal protection, in association with ACEIs or ARBs in patients with GFR ≥ 25 mL/min/1.73 m^2^ and serum potassium levels <5.0 mEq/L.


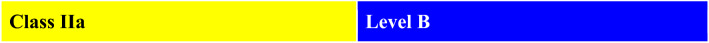


### Summary of evidence

A recent meta-analysis evaluating the nephroprotective effect of mineralocorticoid receptor antagonists (MRAs) assessed 22 studies in patients with DKD and 12 studies in non-diabetic kidney disease. Alone or in combination with RAAS blockers, MRAs reduced the ACR by 24.55% and proteinuria by 53.93% compared to placebo [99].

Spironolactone is associated with a significant reduction in albuminuria. However, side effects, especially in men, such as gynecomastia and sexual dysfunction should not be forgotten [100].

#### R19

The use of non-steroidal mineralocorticoid receptor antagonists MAY BE CONSIDERED for renal protection, in association with ACEIs or BRAs, in patients with GFR ≥ 25 mL/min/1.73 m^2^ , with serum potassium levels <5.0 mEq/L.


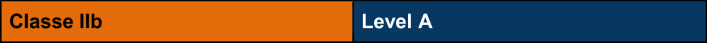


Finerenone is a non-steroidal selective MRA that has been shown to block many of the deleterious effects of overactivation of mineralocorticoid receptors that play an important role in the progression of cardiorenal disease. The complementary studies FIDELIO-DKD and FIGARO-DKD evaluated, in patients with T2DM and CKD, the effect of finerenone on cardiovascular and renal outcomes at different stages of kidney disease.

The FIDELIO-DKD trial randomized 5,734 T2DM patients with DKD (GFR 25–75 mL/min/1.73 m^2^) using ACE inhibitors or ARBs and demonstrated that finerenone was associated with a lower incidence of the composite renal outcome ( sustained drop in eGFR > 40%, progression to dialysis or renal death), with HR 0.82 95% CI of 0.73–0.93, p = 0.001 and number needed to treat (NNT) of 29 patients, in addition to lower incidence of secondary cardiovascular outcome (cardiovascular death, AMI, non-fatal stroke, hospitalization for heart failure), HR 0.86 95% CI 0.75–0.99, p = 0.03 and NNT of 42. In the study, the patient's enrollment potassium level should be ≤ 4.8 mmol/L and checked regularly. In case of increase (> 5.5 mmol/L), the drug was withheld for 72 h, and potassium levels were reassessed. Finerenone was well tolerated, but a slight increase in hyperkalemia was observed (2.3% vs. 0.9%) [101].

In the FIGARO trial, this observation was expanded to 7,437 patients with T2DM and CKD (ACR 30–300 mg/g and eGFR between 25 and 90 mL/min/1.73 m^2^—CKD stages 2–4; or ACR 300–5000 mg /g and eGFR of 60 mL/min/1.73 m^2^ -DRC stages 1–2). Patients on finerenone had a significant decrease in the primary composite CV endpoint (MACE-myocardial infarction, stroke, hospitalization for heart failure, and CV death) (HR, 0.87; 95% CI, 0.76 to 0.98; P = 0.03), with the benefit driven primarily by a lower incidence of hospitalization for heart failure (HR, 0.71; 95% CI, 0.56 to 0.90) compared to placebo. The composite renal endpoint occurred in 350 patients (9.5%) in the finerenone group and in 395 (10.8%) in the placebo group (HR, 0.87; 95% CI, 0.76 to 1.01). Hyperkalemia was four times more frequent in the finerenone group (1.2% vs. 0.4%) [102].

The FIDELITY study was a pre-specified analysis compiling the two studies, FIDELIO and FIGARO. In this analysis, 13,026 participants with a mean follow-up of 3 years were included. Composite cardiovascular outcome occurred in 825 patients (12.7%) in the finerenone group and 939 patients (14.4%) in the placebo group (HR 0.86; 95% CI, 0.78–0.95; p = 0. 0018). The composite renal endpoint occurred in 360 (5.5%) patients in the finerenone group and 465 (7.1%) in the placebo group (HR, 0.77; 95% CI, 0.67–0.88; p = 0 0.0002) with NNT of 60 patients. Hyperkalemia leading to permanent treatment withdrawal was more frequent in the finerenone group (1.7%) than in the placebo group (0.6%). The FIDELITY analysis suggests that finerenone was associated with a significant 20% reduction in ESRD, as well as a reduction in all nonfatal composite renal outcomes [103].

### Treatment of hyperlipidemia in DKD

#### Non-dialytic DKD

##### R20

In patients with DKD and eGFR < 60 mL/min/1.73 m^2^ and post-transplanted patients, the use of high-potency statins IS RECOMMENDED to reduce cardiovascular events.


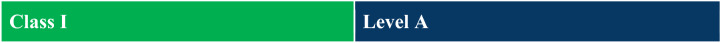


### Summary of evidence

#### Renal risk reduction

In the renal outcomes sub-analysis of the CARDS trial, which randomized 2,838 T2DM individuals without the previous cardiovascular disease to receive either 10 mg atorvastatin or placebo once daily, 34% of patients had eGFR between 30 and 60 mL/min/1.73 m^2^, and 21.5% had albuminuria. Atorvastatin was associated with a modest improvement in albuminuria. Atorvastatin was also associated with an attenuation of eGFR decline (0.18 mL/min/1.73 m^2^/year, 95% CI 0.04–0.32); p = 0.01 but did not change the incidence of new cases of albuminuria or regression to normoalbuminuria [104].

The use of statins decreases the number of cardiovascular events (combined outcome), without decreasing overall or cardiovascular mortality in patients with advanced DKD, regardless of the doses used [105, 106].

In the CARDS study, in 970 patients with eGFR between 30 and 60 mL/min/1.73 m^2^, there was a 42% reduction in major cardiovascular events in the group using 10 mg atorvastatin and a 61% reduction in ischemic stroke, like that observed in the analysis all study patients (37% reduction in cardiovascular events (p = 0.4 interaction) [107].

Statins have a modest effect on albuminuria and the rate of fall in GFR [104, 105] and appear not to affect the rate of hard renal events and progression to renal failure, as suggested in a meta-analysis with more than 143,000 participants [108].

The *National Kidney Foundation* recommends the use of statins to reduce cardiovascular events in patients with pre-dialysis DM [109].

In the SHARP study (*Study of Heart and Renal Protection)* [110], the combination of simvastatin with ezetimibe did not reduce the risk of primary outcomes in dialysis patients. These data indicate that, despite the significant reduction observed in LDL-cholesterol values, the use of statin should occur before the major loss of renal function. It is also not recommended to start statins in dialysis with the aim of primary prevention of cardiovascular events due to loss of efficacy [111]. On the other hand, there is no data to recommend discontinuation of statins when they are already in use before starting dialysis, and, in this situation, their maintenance is suggested [64].

In the case of post-renal transplant patients, in the ALERT study, the use of statins was associated with a lower risk of cardiac death, infarction, and cardiac interventional procedures [112]. Based on these results, the use of statins in patients with T2DM after kidney transplantation is also recommended [113].

The use of fibrates may be associated with a slight decrease in eGFR (a transitory effect that is reversed with the discontinuation of the drug), which is not secondary to kidney damage [113]. Regarding the effect on albuminuria, fibrates offer little benefit in patients with DKD [114, 115]. Therefore, fibrates should only be used in the case of very high triglycerides (>880 mg/dL) to reduce the risk of acute pancreatitis. The need to adjust doses according to renal function is highlighted [116].

The risk of developing myopathy increases with loss of kidney function and in combination with statins, especially gemfibrozil. The use of fenofibrate should be avoided if GFR <30 mL/min/1.73 m^2^.

**Important note 12**: LDLc in DKD.

In patients with CKD, KDIGO recommends that LDL-c values be used to calculate cardiovascular risk, but no longer to decide on the use of lipid-lowering drugs. This recommendation is reinforced, as in patients with CKD, the relationship between LDL-c levels and cardiovascular events seems to be different from the general population [116].

### Patients with DKD on dialysis

#### R21

In patients with DKD on dialysis, without clinical arterial disease, IT IS NOT RECOMMENDED to start using statins. However, in patients who were already using a statin before starting dialysis, it should be continued.


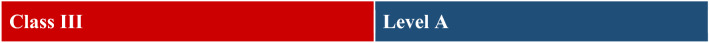


### Summary of evidence

In the 4D study (Die *Deutsch Diabetes Dialyze*) [117], patients with T2DM undergoing hemodialysis were evaluated, 22% of whom had coronary artery disease (CAD). They were randomized to atorvastatin 20 mg or placebo and were followed for four years. The primary endpoint was a composite of death from cardiac causes, non-fatal acute myocardial infarction, and stroke. A 42% reduction in LDL-c was observed in patients on atorvastatin with no reduction in the primary endpoint. The risk of stroke was also higher in this group.

The AURORA study (Rosuvastatin *and cardiovascular events in patients with regular hemodialysis*) [118] included 2776 patients on hemodialysis (aged 50–80 years, 27.9% with diabetes and 39% with CAD) treated with rosuvastatin 10 mg/day or placebo for 3.8 years on average. The primary endpoint was a composite of non-fatal myocardial infarction, non-fatal stroke, and cardiovascular death. There was a 43% reduction in LDL-c in the intervention group, but no difference in the primary outcome was observed between the groups.

For patients with CKD, but not on hemodialysis, the *Pravastatin Pooling Project* database performed a pooled analysis of the results of three randomized trials with pravastatin 40 mg vs. placebo, including 19,700 patients with chronic renal failure (eGFR 60–30 mL/min/1.73 m^2^) [57]. There was a significant benefit of treatment in reducing the primary outcome of myocardial infarction, coronary death, or percutaneous revascularization and total mortality in this group of patients [119].

Neither atorvastatin nor rosuvastatin reduced cardiovascular mortality, infarction, and/or stroke in hemodialysis patients [117, 118]. However, in a *post hoc* analysis of 731 patients with T2DM, a reduction in the risk of fatal and non-fatal cardiac events was observed with the use of rosuvastatin [120].

The SHARP study (The *effects of lowering LDL cholesterol with simvastatin plus ezetimibe in patients with chronic kidney disease*) [110] aimed to evaluate the efficacy and safety of the combination of simvastatin plus ezetimibe in subjects with moderate to severe CKD. This is a randomized, double-blind study that included 9,270 patients with CKD (3,023 on dialysis and 6,247 not on dialysis), with no known history of myocardial infarction or coronary revascularization. Patients were randomized to simvastatin 20 mg plus ezetimibe 10 mg daily *vs*. placebo of the two medications. The group allocated to the simvastatin and ezetimibe had a mean LDL-c reduction of 33 mg/dL during a mean follow-up of 4.9 years. There was a 17% proportional reduction for major atherosclerotic events for simvastatin plus ezetimibe compared to placebo (RR 0.83; 95% CI 0.74–0.94; p = 0.0021).

A sub-analysis of the TNT study assessed how intensive lipid lowering with 80 mg of atorvastatin would affect renal function, compared with 10 mg, in patients with coronary heart disease. A total of 10,001 patients with coronary heart disease and LDL-c levels < 130 mg/dL were randomized in a double-blind fashion to 10 mg/day or 80 mg/day atorvastatin therapy. The GFR estimated using the MDRD (*Modification of Diet in Renal Disease*) equation was compared at the beginning and end of follow-up in 9,656 participants. No decline in kidney function was observed over five years. In contrast, estimated GFR improved in both treatment groups and was significantly higher at the 80 mg dose, suggesting that such benefit may be related to medication dosage [121].

#### R22

In patients on hemodialysis and LDL-c above 145 mg/dL and/or with established coronary artery disease, statin initiation MAY BE CONSIDERED.


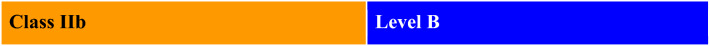


### Summary of evidence

In a *posthoc* analysis of 731 patients with T2DM, a reduction in the risk of fatal and non-fatal cardiac events was observed with the use of rosuvastatin ref [120, 115]. Based on a subgroup analysis of the 4D study, in which patients with LDL above 145 mg/dL benefited from reduced cardiovascular mortality, non-fatal AMI, death from any cause, and sudden death [117].

### Nutrition therapy

#### R23

For individuals with non-dialysis-dependent advanced CKD, it is RECOMMENDED a dietary protein intake of around 0.8 g/kg ideal body weight per day.


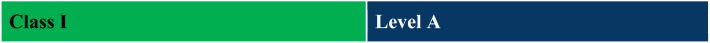


### Summary of evidence

Dietary protein restriction has been suggested to patients with CKD from many etiologies. However, low adherence to this dietary intervention is observed, especially because it implies a change in lifestyle habits. Due to the lack of consensus in the literature about the benefit of dietary protein restriction in patients with DM and increased UAE, but preserved eGFR, there is no specific recommendation for these patients [122, 123].

In patients with increased UAE and decreased eGFR, moderate dietary protein restriction (0.8 g/kg ideal weight/day) is recommended [22].

Protein intake above 20% of daily calories or above 1.3 g/kg ideal weight/day is associated with increased albuminuria, more rapid loss of renal function, and CV mortality; therefore, it should be avoided. A meta-analysis with 779 patients from 13 RCTs demonstrated a benefit from a low-protein diet with improved eGFR and reduced proteinuria [124, 125].

The effect of dietary protein restriction becomes more evident the greater the adherence to dietary modification and, when RAAS inhibitors are used, it is less frequent and the BP control less strict [126].

A high protein intake is associated with an increase in glomerular filtration rate, serum urea, acid uric acid, and urinary calcium excretion when compared to a normal/low protein intake in normal subjects, as reported in a meta-analysis including 30 randomized controlled trials [127].

Therefore, the rationale for protein restriction in the CKD setting is based on the decrease in kidney overload, which leads to an improvement in renal hemodynamics by decreasing the intraglomerular pressure and glomerular hyperfiltration. It is also a key strategy to control uremia and uremic toxins, as well as oxidative stress, metabolic acidosis, phosphorous, hyperparathyroidism, insulin resistance, and blood pressure [128].

According to KDOQI guidelines, for CKD 3–5 patients not on dialysis and without DM, it is recommended a low-protein diet providing 0.55–0.60 g dietary protein/kg body weight/day, or a very low-protein diet providing 0.28–0.43 g dietary protein/kg body weight/day with additional keto acid/amino acid analogs to meet protein requirements (0.55–0.60 g /kg body weight/day), as these approaches halt the progression of CKD (1A) and improve the quality of life (2C) [129].

Likewise, for CKD 3–5 patients not on dialysis but with DM, it is suggested a dietary protein intake of 0.6–0.8 g/kg body weight per day to maintain a stable nutritional status and optimize glycemic control to avoid CKD progression (opinion) [129]. Thus, the impact of protein restriction warrants further investigation.

For CKD patients under chronic hemodialysis (1C) and peritoneal dialysis (opinion) without DM, it is recommended to prescribe a dietary protein intake of 1.0–1.2 g/kg body weight per day to maintain a stable nutritional status [129]. For those patients with DM under maintenance hemodialysis and peritoneal dialysis, it is suggested to prescribe a dietary protein intake of 1.0–1.2 g/kg body weight per day to maintain a stable nutritional status (opinion). For patients at risk of hyperglycemia and/or hypoglycemia, higher levels of dietary protein intake may need to be indicated to maintain glycemic control (opinion) [128].

To note, protein restriction should be prescribed under close clinical supervision to avoid inadequate caloric intake, protein loss and hypercatabolism, inflammation, and altered glucose homeostasis [128].

Importantly, the impact of a very low-protein diet on kidney disease progression is a subject of debate in the literature. The Modification of Diet in Renal Disease (MDRD) study evaluated the effect of two schedules of protein restriction in patients with nondiabetic CKD 4. When the intervention of a low-protein diet (0.58 g/kg/d) was compared to a very low-protein diet (0.28 g/kg/d) supplemented with a mixture of essential keto acids and amino acids (0.28 g/kg/d), the latter group presented higher rates of mortality and no beneficial effect on CKD progression was observed [130].

**Important note 13:** Meat consumption and Diabetic Kidney Disease.

An alternative is to replace red meat with other protein sources. A diet where red meat was replaced by chicken, rich in polyunsaturated fatty acids, was able to decrease the UAE in T2DM patients with micro and macroalbuminuria [131, 132].

#### R24

The limit for a sodium intake of up to 1.5 g/day, or of salt, up to 3.75 g/day, SHOULD BE CONSIDERED when there is arterial hypertension.


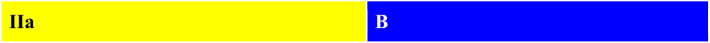


### Summary of evidence

When making dietary recommendations for patients with DKD, it should be considered that, in most cases, there is associated hypertension. Thus, limiting dietary salt intake should be a goal to be achieved.

Decreasing dietary salt enhances the antihypertensive effect of drugs [133]. Furthermore, the renal and cardiovascular effects of ARBs are enhanced when associated with the restriction of salt intake [53].

Salt restriction should be included in a DASH (Dietary Approaches to Stop Hypertension) diet pattern, that is, high consumption of fruits, vegetables, and low-fat dairy products. In T2DM patients, this dietary pattern is associated with lower blood pressure values. This diet, however, is not recommended for dialysis patients [134].

## Conclusions

Diabetic kidney disease (DKD) is the leading cause of entry into renal replacement therapy and is associated with increased morbidity and mortality. Traditionally, DKD is defined as a sequential evolution of stages characterized by a progressive increase in urinary albumin excretion (UAE) between 30 mg/day and 300 mg/day (formerly known as microalbuminuria) and followed by UAE levels greater than 300 mg/day or macroalbuminuria. In this phase, a progressive loss of glomerular filtration rate (GFR) starts, culminating in end-stage renal failure. In recent years it has been recognized that this evolution does not always happen, as there are patients who lose glomerular filtration without developing albuminuria, a fact associated with multiple factors such as hypertension, dyslipidemia, obesity, and age. To prevent or at least postpone the advanced stages of DKD with the associated cardiovascular complications, intensive glycemic and blood pressure control are required, as well as the use of renin–angiotensin–aldosterone system blocker agents such as ARB, ACEI, and MRA. Recently, SGLT2 inhibitors and GLP1 receptor agonists have been added to the therapeutic arsenal, with well-proven benefits regarding kidney protection and patients’ survival.

**Table 3:**
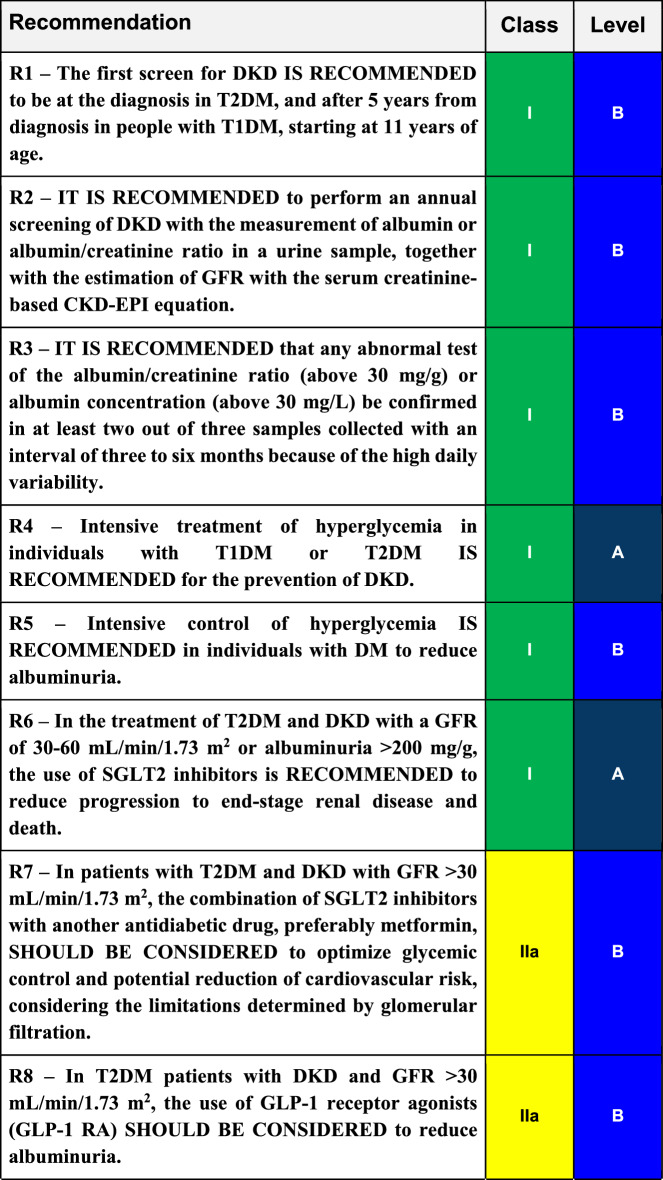

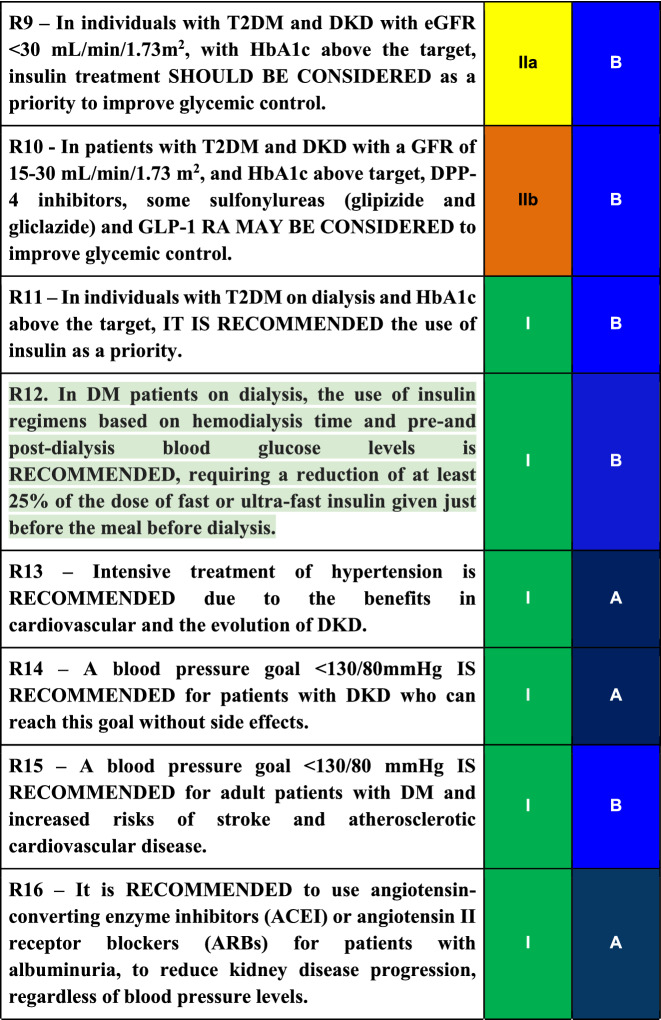

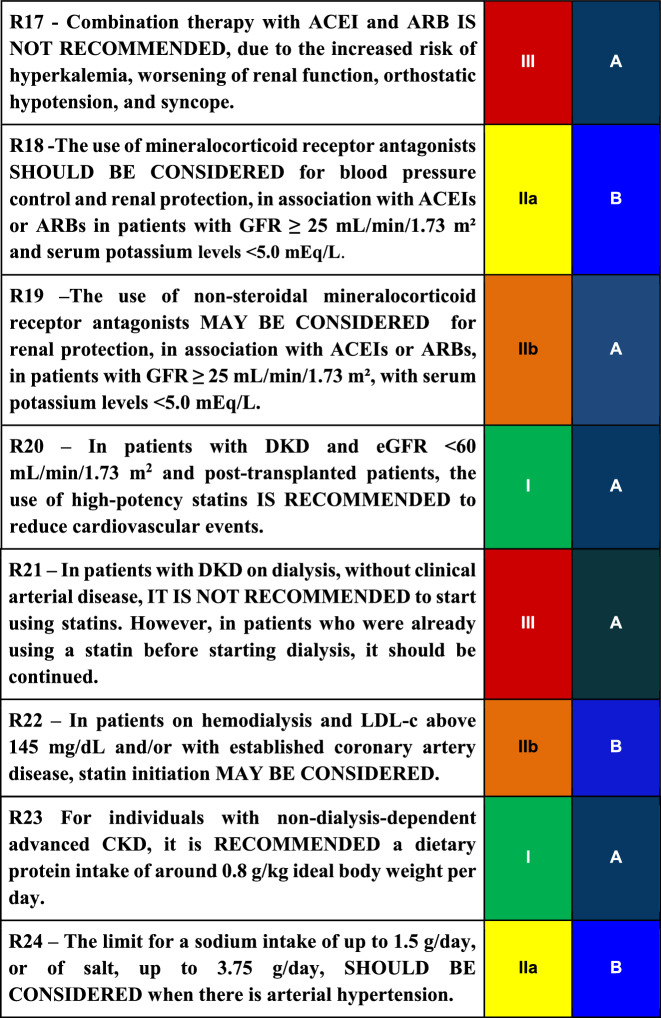
Final recommendations

## Data Availability

Data sharing does not apply to this article.

## Abbreviations

ACE: Angiotensin-converting enzyme inhibitor
ADA: American Diabetes Association
AMI: Acute myocardial infarction
ARB: Angiotensin receptor blocker
BP: Blood pressure
CKD: Chronic Kidney Disease
CKD-EPI: Chronic Kidney Disease Epidemiology Collaboration
CV: Cardiovascular
DASH: Dietary Approaches to Stop Hypertension
DKD: Diabetic kidney disease
DM: Diabetes mellitus
DPP4: Dipeptidyl peptidase 4
GA: Glycated albumin
GFR: Glomerular filtration rate
GLP1 RA: Glucagon-like peptide 1 receptor agonist
KDIGO: Kidney Disease Improving Global Outcomes
KDOQI: Kidney Disease Outcomes Quality Initiative
RAAS: Renin–angiotensin–aldosterone system
RCT: Randomized controlled trials
SBD: Brazilian Society of Diabetes
SGLT2i: Sodium-glucose transport 2 inhibitors
type 1 DM: T1DM
type 2 DM: T2DM
UAE: Urinary albumin excretion
